# Iowa District School Journal

**Published:** 1853-10

**Authors:** 


Iowa District School Journal.—This excellent work is ably edited by our friend Dr. Gilbert, of Dubuque, in a manner which reflects great credit upon his ability and capacity; from the utility of such a work and the neatness with which it is executed, it is an honor to the State, and deserves to be liberally patronized.

Physicians are, or should be, the friends of education;—they should be zealous laborers, to advance the interests of education, wherever they are, and to the utmost of their influence; and they will find that their influence is much, if they will but exert it. Generous impulses and patriotic sentiments would suggest and encourage such a duty; but if these are wanting, the commonest views of selfishness would stimulate to efforts to raise the standard of public intelligence; for nothing so much interferes with a physician's success, embarrasses his efforts, or increases his labors, as ignorance and superstition. Inform us of the number of churches and schools in a city or village of known population, and we can very easily estimate the character of the medical profession in that region. For ignorance, with its attendant evils, constitutes the *life-blood* of quacks; if this does not exist in a community, they can no longer exercise the low cunning by which they mislead the judgment, and impose upon the credulities of others no better informed, but more honest than themselves.

We would especially urge the physicians of this young and growing State, to lend a helping hand to the noble cause of public education. There are numerous ways in which this may be done—by public lectures upon topics of general interest—by written articles, in appropriate papers and journals—by serving faithfully and jealously, upon school committees—at the very least, by patronizing the State School Journal at Dubuque, and in every way which an anxiety to do good will readily suggest. There is no more worthy field of labor—there is none (out of his own strict line of practice) in which he can be of more lasting service to the community in which and for which he lives.

